# IRAK1 inhibition blocks the HIV-1 RNA mediated pro-inflammatory cytokine response from microglia

**DOI:** 10.1099/jgv.0.001858

**Published:** 2023-05-31

**Authors:** Grant R. Campbell, Pratima Rawat, Carmen Teodorof-Diedrich, Stephen A. Spector

**Affiliations:** ^1^​ Division of Basic Biomedical Sciences, Sanford School of Medicine, University of South Dakota, Vermillion, SD, USA; ^2^​ Division of Infectious Diseases, Department of Pediatrics, University of California San Diego, La Jolla, CA, USA; ^3^​ Rady Children’s Hospital, San Diego, CA, USA; ^†^​ Present address: Microbiologics Inc, San Diego, CA, USA

**Keywords:** HIV, IRAK1, microglia, pacritinib, TLR7, TLR8

## Abstract

Human immunodeficiency virus (HIV)-associated neurocognitive disorders (HAND) are a common source of morbidity in people living with HIV (PLWH). Although antiretroviral therapy (ART) has lessened the severity of neurocognitive disorders, cognitive impairment still occurs in PLWH receiving ART. The pathogenesis of HAND is likely multifaceted, but common factors include the persistence of HIV transcription within the central nervous system, higher levels of pro-inflammatory cytokines in the cerebrospinal fluid, and the presence of activated microglia. Toll-like receptor (TLR) 7 and TLR8 are innate pathogen recognition receptors located in microglia and other immune and non-immune cells that can recognise HIV RNA and trigger pro-inflammatory responses. IL-1 receptor-associated kinase (IRAK) 1 is key to these signalling pathways. Here, we show that IRAK1 inhibition inhibits the TLR7 and TLR8-dependent pro-inflammatory response to HIV RNA. Using genetic and pharmacological inhibition, we demonstrate that inhibition of IRAK1 prevents IRAK1 phosphorylation and ubiquitination, and the subsequent recruitment of TRAF6 and the TAK1 complex to IRAK1, resulting in the inhibition of downstream signalling and the suppression of pro-inflammatory cytokine and chemokine release.

## Introduction

Although advances in antiretroviral therapy (ART) have vastly reduced morbidity and mortality of people living with human immunodeficiency virus (HIV) type 1, a spectrum of neurocognitive impairments (NCIs) collectively referred to as HIV-associated neurocognitive disorders (HAND) are still prevalent in ~40 % of people living with HIV (PLWH) on suppressive ART [1, 2]. The clinical severity of NCIs in HAND ranges from asymptomatic (asymptomatic NCI) to severe (HIV-associated dementia). The pathogenesis of HAND is not fully understood and is likely multifactorial, but common factors are the presence of HIV RNA transcripts within the central nervous system (CNS) [3–5], chronic inflammation and immune activation [6].

Microglia are CNS resident macrophages that mediate the innate and adaptive immune and repair responses to invading pathogens and injury through the recognition of pathogen-associated molecular patterns (PAMPs) like viral proteins and nucleic acids by innate pattern recognition receptors (PRR) such as Toll-like receptors (TLRs) [7]. The ligation of PAMPs to TLRs leads to the transcription and release of pro-inflammatory cytokines and chemokines. Microglia are an important source of these PAMPs within the CNS of PLWH [8–13].

HIV RNA transcripts can be hydrolytically degraded by lysosomal endoribonucleases generating guanosine/uridine-rich ssRNA fragments that are sensed by endosomal TLR7 and TLR8 [14–18]. The binding of GU-rich ssRNA to TLR7 or TLR8 induces receptor dimerisation and the recruitment of the adaptor protein MYD88 and IL-1 receptor-associated kinase (IRAK) 4, IRAK1, IRAK2 and/or IRAK3, forming the myddosome that results in the activation and phosphorylation of IRAK1 by IRAK4 [19–23]. Activated IRAK1 then phosphorylates and activates pellino E3 ubiquitin protein ligases 1 and 2 leading to the recruitment and activation of the E3 ubiquitin ligase TNF receptor-associated factor 6 (TRAF6), as well as the lysine 63 (K63) and Met1 (M1) linked ubiquitination of IRAK1 [24–26]. The ubiquitinated IRAK1–TRAF6 complex is then released from the myddosome where it can activate the TAK1 and IKK complexes that then activate a number of downstream pro-inflammatory mediators, including mitogen activating protein kinases and transcription factors, resulting in the expression of pro-inflammatory cytokines [27–29].

The role of microglia TLR7 and TLR8 in HAND is unknown. However, activation of TLR7 in murine models leads to increased *TLR8* expression, microglia activation, neuroinflammation, and neuronal cell death [30, 31]. Additionally, human microglia exposed to HIV RNA secrete IL-1α, IL-1β, IL-18, TNF, ROS, and C1QA [32]. As IRAKs are critical for TLR-mediated transcription and release of pro-inflammatory cytokines, inhibiting IRAK1 may have potential therapeutic benefits in HIV disease [33–36]. Pacritinib is a small-molecule, ATP-competitive, macrocyclic inhibitor of JAK2 (IC_50_ 23 nM), FMS-like tyrosine kinase 3 (FLT3; IC_50_ 22 nM), IRAK1 (IC_50_ 6 nM), and IRAK4 (IC_50_ 177 nM) [36, 37] that has been FDA (Food and Drug Administration, USA) approved for the treatment of myelofibrosis marked by severe thrombocytopaenia. Here, we show that pacritinib inhibits HIV RNA mediated pro-inflammatory cytokine and chemokine responses from microglia.

## Methods

### Microglia

Peripheral blood mononuclear cells (PBMCs) were isolated from whole blood obtained from Innovative Research, the San Diego Blood Bank, or drawn from human subjects at the University of California San Diego Health Sciences by density gradient centrifugation over Ficoll-Paque Plus (Cytiva). Human monocyte-derived microglia (MMG) were differentiated from primary human monocytes as described elsewhere [38–41]. Briefly, 6×10^6^ PBMCs ml^−1^ were incubated in MMG media [RPMI 1640 Glutamax supplemented with 100 µg streptomycin ml^−1^, 100 U penicillin ml^−1^ (all Gibco), 10 ng CSF1, CSF2, and β-NGF ml^−1^, and 100 ng CCL2 and IL-34 ml^−1^ (all Peprotech)] for 4 h, after which non-adherent cells were removed by aspiration. Adherent cells were then washed with Dulbecco’s PBS (Gibco) and further incubated in MMG media for 14 days at humidified 37 °C, 5 % CO_2_ with media changes every 3 days, at which point cells were cultured in RPMI 1640 Glutamax supplemented with 100 µg streptomycin ml^−1^ and 100 U penicillin ml^−1^ .

### HIV

HIV Ba-L (ARP-510) was obtained through the National Institutes of Health (NIH) AIDS Reagent Program from Suzanne Gartner, Mikulas Popovic and Robert Gallo [42, 43]. Virus stocks were prepared, purified, and concentrated as previously described [44]. Total viral RNA was extracted directly from virus concentrates using the QIAamp viral RNA mini kit (Qiagen), quantified using a NanoDrop instrument (Thermo Fisher), and complexed with Lipofectamine MessengerMAX (Thermo Fisher) according to the manufacturer’s instructions. HIV Gag-iGFP_JRFL (ARP-12456) was obtained through the NIH AIDS Reagent Program from Benjamin Chen. A total of 2×10^4^ 293T cells cm^−2^ (ATCC CRL-3216) were plated in T75 flasks for 24 h, washed, then transfected with 10 µg HIV-Gag-iGFP_JRFL using GeneJuice (EMD Millipore) according to the manufacturer's protocol, washed, then further incubated for 48 h at humidified 37 °C, 5 % CO_2_ before use. Infection was assessed by flow cytometry after trypsinisation for 10 mins, followed by fixation with 4 % (v/v) paraformaldehyde for 25 min. Data were acquired on a BD FACSCalibur flow cytometer with CellQuest Pro v. 5.2.1 acquisition software (BD Biosciences) using the 488 nm blue laser for excitation and a 530 nm/30 nm bandpass filter for detection of GFP emission. Analysis was performed using FlowJo v10.9.0.

### Chemicals

RNA40 [5′-GCCCGUCUGUUGUGUGACUC-3′; at U5 region 108–127 nt of the HIV genome (accession no. NC_001802.1)] [14], RNA41 (a derivative of RNA40 in which adenosine replaces all uracil nucleotides), RNA649 [5′-GUCAGAGUGUGUACUUG-3′; position 24 649–24 665 nt in the SARS-CoV-2 genome (S2 spike protein region) (accession no. NC_045512.2)] [16], RNA649A (a derivative of RNA649 in which adenosine replaces all uracil nucleotides), IRS 661 (5′-TGCTTGCAAGCTTGCAAGCA-3′) [45] and ODN (5′-TCCTGCAGGTTAAGT-3′) [45] were synthesised by Integrated DNA Technologies. LyoVec (InvivoGen), a cationic lipid-based transfection reagent, was used to complex GU-rich ssRNA in a 2 : 1 (LyoVec:RNA) ratio as previously described [16]. RNA40 and RNA41 precomplexed with LyoVec were purchased from InvivoGen. Recombinant human ubiquitin specific peptidase 2 (USP2) and CU-CPT4a were purchased from R&D Systems. CU-CPT9a, JH-X-119-01, maraviroc, pacritinib (SB1518), plerixafor, and zimlovisertib were purchased from Selleck Chemicals. Lambda protein phosphatase (λ-PPase) and MG132 were purchased from Sigma-Aldrich.

### Cell death and inflammatory markers

Lactate dehydrogenase (LDH) activity of supernatants was measured using the LDH cytotoxicity detection kit (Takara Bio) and per cent cytotoxicity calculated according to the manufacturer’s protocol. Cell supernatants were also assayed for the presence of chemokines and cytokines using ELISA kits obtained from R&D Systems: IFNα (catalogue no. DFNAS0), IL-1β (catalogue no. DLB50 and DY201), IL-6 (catalogue no. DY206), IL-8 (catalogue no. DY208), CXCL10 (catalogue no. DY266), CCL2 (catalogue no. DY279) and TNF (catalogue no. DY207).

### Small interfering RNA (siRNA) transfection

MMG were transfected with Ambion Silencer Select *P2RX7* (ID no. s9959), *TLR2* (ID no. s76898), *TLR4* (ID no. s14195), *TLR7* (ID no. s27842), *TLR8* (ID no. s27920), or control (catalogue no. 4390846) siRNA (siNS) using lipofectamine RNAiMAX (Invitrogen) in Opti-MEM (Gibco) according to the manufacturer’s instructions. Cells were analysed for target gene silencing 48 h post-transfection and used in experiments. Transfection efficiency was assessed using BLOCK-iT Alexa Fluor red fluorescent control (Invitrogen) on a Countess 3 FL automated cell counter (Thermo Fisher).

### Western blotting

Cell lysis, co-immunoprecipitation, and western blotting were performed as previously described [44, 46]. Briefly, cell lysates were prepared using 20 mM HEPES, 150 mM NaCl, and 1 mM EDTA supplemented with 1 % (v/v) Triton X-100 and 1 % (v/v) Thermo Scientific Halt protease and phosphatase inhibitor cocktail. Co-immunoprecipitation was performed using the Pierce co-immunoprecipitation kit with 50 µg anti-IRAK1 (cataologue no. ab180747; RRID AB_2895218) from Cell Signaling and 50 µg cell lysates, according to the manufacturer’s directions. Incubation of immunoprecipitates with phosphatases and deubiquitinases was performed as described elsewhere [23, 47]. Cell lysates and immunoprecipitates were resolved using 2-[bis(2-hydroxyethyl)amino]−2-(hydroxymethyl)propane-1,3-diol buffered (w/v) polyacrylamide gels in MOPS buffer, transferred to 0.45 µm nitrocellulose membranes, probed with primary antibodies overnight at 4 °C followed by detection using alkaline phosphatase tagged secondary antibodies (Invitrogen) and 0.25 mM CDP-Star supplemented with 5 % (v/v) Nitro-Block II (both Applied Biosystems). The primary antibodies raised against the following were used in western blots: ERK1/2 (catalogue no. 4695, RRID AB_ 390779), phospho-ERK1/2 (Thr^202^/Tyr^204^) (catalogue no. 4370, RRID AB_2315112), IκBα (catalogue no. 4812, RRID AB_10694416), phospho-IκBα (Ser^32^) (catalogue no. 2859, RRID AB_561111), IL-1β (catalogue no. 12703, RRID AB_2737350), IRAK1 (catalogue no. 4504, RRID AB_1904032), IRAK2 (catalogue no. 4367, RRID AB_2126268), IRAK3 (catalogue no. 4369, RRID AB_2264868), IRAK4 (catalogue no. 4363, RRID AB_2126429), IRF5 (catalogue no. 20261, RRID AB_ 2798838), JNK (catalogue no. 9252, RRID AB_2250373), phospho-JNK (Thr^183^/Tyr^185^) (catalogue no. 4671, RRID AB_331338), NLRP3 (catalogue no. 13158, RRID AB_2798134), P2RX7 (catalogue no. 13809, RRID AB_ 2798319), p38 MAPK (catalogue no. 9212, RRID AB_330713), phospho-p38 MAPK (Thr^180^/Tyr^182^) (catalogue no. 4511, RRID AB_2139682), TAB2 (catalogue no. 3745, RRID AB_2297368), TAK1 (catalogue no. 5206, RRID AB_10694079), TLR2 (catalogue no. 12276, RRID AB_2797867), TLR4 (catalogue no. 38519, RRID AB_2924306), TLR7 (catalogue no. 5632, RRID AB_10692895), and TRAF6 (catalogue no. 67591, RRID AB_2904212) from Cell Signaling Technology, β-actin (ACTB) (catalogue no. A2228, RRID AB_476697) from Sigma-Aldrich, phospho-IRAK1 (Thr^209^) (catalogue no. PA5-38633, RRID AB_2555228) from Invitrogen, TLR8 (catalogue no. NBP2-24917, RRID AB_2847894) from Novus Biologicals, and phospho-IRF5 (Ser^462^) from the MRC Protein Phosphorylation and Ubiquitylation Unit at the University of Dundee, Scotland, UK (catalogue no. IRF5 S509D, RRID AB_2905520). The relative densities of the target bands were compared to the reference bands (ACTB) using Fiji from the Max Planck Institute of Molecular Cell Biology and Genetics (Fiji, RRID SCR_002285) [48].

### Statistics

Samples were assigned to experimental groups through simple random sampling. Sample size (*n*) was determined using a two-sample two-sided equality test with power (1*−β*) = 0.8, *α*=0.05 and preliminary data where the minimum difference in outcome was at least 70 %. Data are represented as dot blots with arithmetic means±sd. Data were assessed for symmetry and skewness using Pearson’s skewness coefficient. Normalized ratiometric data were log_2_ transformed. When protein expression in the reference sample was below the threshold for detection, we used proportion statistics to analyse differences [44, 49]. Comparisons between groups were performed using the paired, two-tailed, Student’s *t*-test. In all experiments, differences were considered significant when *P*<0.05.

## Results

### HIV-derived GU-rich ssRNA induces TLR7 and TLR8-mediated phosphorylation and ubiquitination of IRAK1 in human microglia

**Fig. 1.**
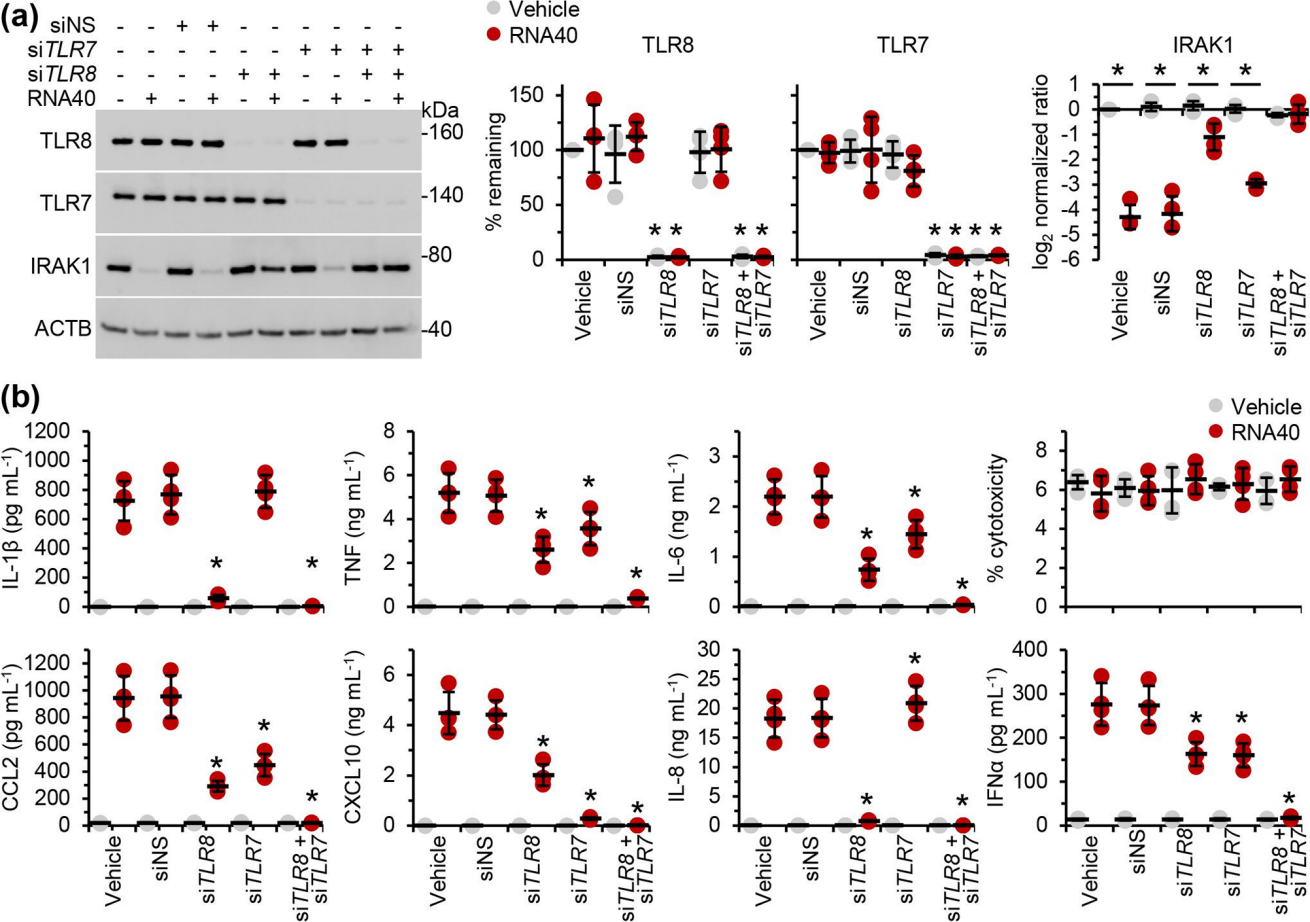
RNA40 induces the disappearance of IRAK1 from western blots and the release of pro-inflammatory cytokines from microglia through both TLR7 and TLR8. MMG were transfected with *TLR*7 (si*TLR7*), *TLR8* (si*TLR8*), or scrambled siRNA (siNS) for 48 h then treated with 5 µg RNA40 ml^−1^ or controls for 24 h. Cells and supernatants were then harvested. (**a**) Cells were lysed and analysed for TLR7, TLR8, and IRAK1 expression levels. *Left*, representative western blots of TLR7, TLR8, IRAK1, and ACTB (the internal reference) from one biological replicate. *Right*, densitometric analysis of blots. Graphs represent the relative intensity of each target band to the corresponding ACTB signal. Each symbol represents an individual biological replicate (*n*=4); small horizontal lines indicate the means of four biological replicates±sd. (**b**) Cell supernatants were analysed for cytokines and chemokines by ELISA, and LDH release using an LDH activity assay as a measure of cytotoxicity (% cytotoxicity). Each symbol represents the mean of an individual biological replicate performed in triplicate; small horizontal lines indicate the grand means of four biological replicates±sd. Statistical analyses were performed using two-tailed paired *t*-tests; *P<0.05.

HIV is a retrovirus with two copies of a ssRNA genome that has been shown to elicit an autophagic and pro-inflammatory response from human macrophages and CD4^+^ T cells via endosomal TLRs [14, 16, 47, 50–52]. We previously demonstrated that RNA40 (a GU-rich ssRNA sequence derived from the HIV LTR [14]) elicits TLR8-dependent autophagic and pro-inflammatory cytokine responses from primary human macrophages [16, 53]. To assess the ability of RNA40 to elicit a TLR8-dependent response from human microglia, we silenced *TLR8* in human MMG then treated the cells with RNA40 (Fig. 1a). Unlike that which we observed in human macrophages [16], *TLR8* silencing did not result in complete inhibition of the RNA40 driven pro-inflammatory cytokine and chemokine responses from MMG (Fig. 1b). However, *TLR8* silencing did completely inhibit the production of IL-1β and IL-8. We also tested the role of TLR7 using RNAi. Similarly, *TLR7* silencing failed to completely inhibit the secretion of pro-inflammatory cytokines and chemokines in response to RNA40; however, it did have a significant effect on CXCL10 (IP-10) production. Importantly, while silencing either of *TLR7* or *TLR8* only resulted in a slight reduction in TNF (TNFα), IL-6, CCL2 (MCP-1), and total IFNα production in response to RNA40, silencing both simultaneously completely inhibited their secretion (Fig. 1b).

**Fig. 2.**
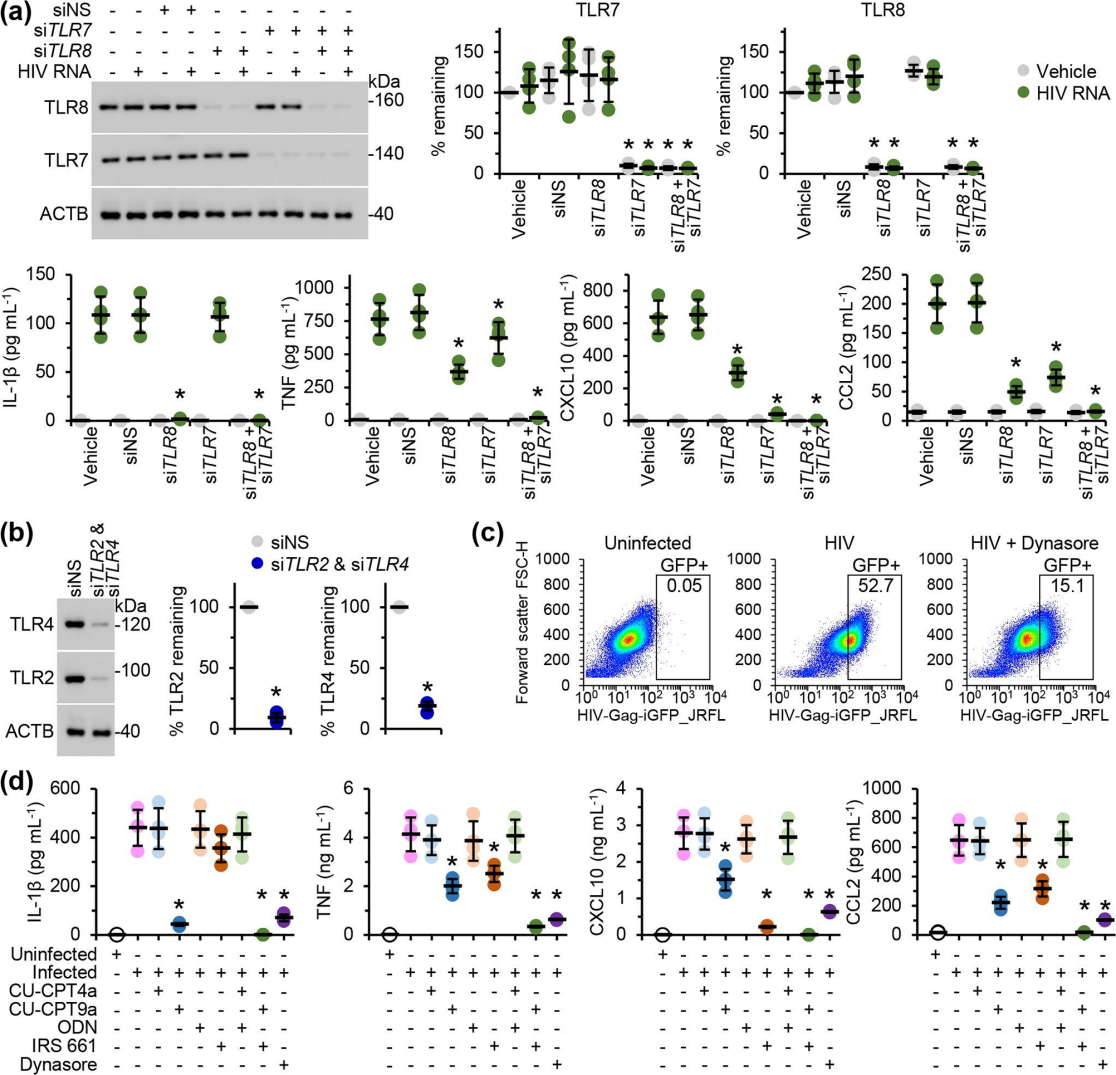
HIV induces the release of pro-inflammatory cytokines from MMG through both TLR7 and TLR8. (**a**) MMG were transfected with *TLR*7 (si*TLR7*) and/or *TLR8* (si*TLR8*), or scrambled siRNA (siNS) for 48 h then treated with 1 µg HIV RNA ml^−1^ for 24 h, after which cells were lysed and supernatants harvested. *Top left*, representative western blots of TLR7, TLR8, and ACTB (the internal reference) from one biological replicate. *Top right*, densitometric analysis of blots. Graphs represent the relative intensity of each target band to the corresponding ACTB signal. Each symbol represents an individual biological replicate (*n*=4); small horizontal lines indicate the means of four biological replicates±sd. *Bottom*, cell supernatants were analysed for cytokines and chemokines by ELISA. Each symbol represents the mean of an individual biological replicate performed in triplicate; small horizontal lines indicate the grand means of four biological replicates±sd. (**b**) MMG were simultaneously silenced for *TLR*2 (si*TLR2*) and *TLR4* (si*TLR4*) for 48 h, after which cells were lysed and analysed for TLR2 and TLR4 expression levels. *Left*, representative western blots of TLR2, TLR4, and ACTB (the internal reference) from one biological replicate. *Right*, densitometric analysis of blots. Graphs represent the relative intensity of each target band to the corresponding ACTB signal. Each symbol represents an individual biological replicate (*n*=4); small horizontal lines indicate the means of four biological replicates±sd. (**c**) MMG from (b) were pre-treated with 5 µg maraviroc ml^−1^ and 5 µg plerixafor ml^−1^ for 24 h and 80 µM dynasore for 1 h, washed, then co-cultured with 293T cells expressing HIV Gag-iGFP_JRFL for a further 24 h in the presence of maraviroc and plerixafor. Cultures were trypsinised and MMG purified using CD11b microbeads, followed by analysis by imaging flow cytometry. Pseudocolour plots from a single biological replicate show frequencies of trypsinised GFP-positive MMG. (**d**) MMG from (b) were pre-treated with 5 µg plerixafor ml^−1^ and 5 µg maraviroc ml^−1^ for 24 h and 25 µM CU-CPT9a (a selective TLR8 antagonist), 25 µM CU-CPT4a (a selective TLR3 inhibitor and negative control for CU-CPT9a), 5.6 µM IRS 661 (a selective TLR7 antagonist), 5.6 µM ODN (a negative control for IRS 661) for 2 h, or 80 µM dynasore for 1 h prior to co-culture with 293T cells expressing HIV Gag-iGFP_JRFL for a further 24 h in the presence of all inhibitors/controls except dynasore. Cell supernatants were then analysed for cytokines and chemokines by ELISA. Each symbol represents the mean of an individual biological replicate performed in triplicate; small horizontal lines indicate the grand means of four biological replicates±sd. Statistical analyses were performed using two-tailed paired *t*-tests; *P<0.05.

To assess whether genomic HIV RNA also triggers a pro-inflammatory cytokine response via TLR7 or TLR8 signalling in human microglia, MMG silenced for *TLR7*, *TLR8*, or both *TLR7* and *TLR8* were incubated for 24 h with HIV RNA isolated from viral particles, after which IL-1β, CXCL10, TNF, and CCL2 were analysed by ELISA. Similar to RNA40, the HIV RNA-induced IL-1β response was dependent upon TLR8, CXCL10 was dependent upon TLR7, and the TNF and CCL2 responses were dependent upon both (Fig. 2a). We then examined whether HIV is sensed by TLR7 or TLR8 in the context of viral infection using an established model of cell-to-cell transmission [52]. We initially co-cultured *TLR2* and *TLR4* silenced MMG (Fig. 2b) with 293T cells expressing R5-tropic HIV-Gag-iGFP_JRFL in the presence of maraviroc (a CCR5 antagonist) and plerixafor (a CXCR4 antagonist) to block fusion and then quantified the frequency of MMG harbouring intracellular HIV using flow cytometry (Fig. 2c). *TLR2* and *TLR4* were silenced as structural and regulatory proteins of HIV have been shown to trigger pro-inflammatory responses through these TLR [54, 55]. For these experiments, we did not use RNAi for TLR7 and TLR8 as we were unable to achieve efficient silencing for all four TLRs simultaneously. Inhibition of HIV uptake by pre-treatment with the dynamin inhibitor dynasore markedly reduced the frequency of MMG harbouring trypsin-resistant GFP, indicating that HIV is efficiently endocytosed upon cell-to-cell transfer in the absence of co-receptor binding. Importantly, as dynasore can interfere with HIV replication [56], MMG were only pre-treated with dynasore prior to co-culture with infected 293T cells, leading to only a partial inhibition of virus transfer. We then analysed cytokine expression by ELISA after 24 h of co-culture in the presence or absence of TLR7 (IRS 661) and/or TLR8 (CU-CPT9a) antagonists or controls (Fig. 2d). Endosomal HIV significantly increased the levels of pro-inflammatory cytokine and chemokine responses, and the TLR8 and/or TLR7 antagonists efficiently reduced or completely inhibited these responses (Fig. 2d). Thus, RNA40 is a suitable surrogate for the study of HIV-induced TLR7 and TLR8 signalling.

**Fig. 3.**
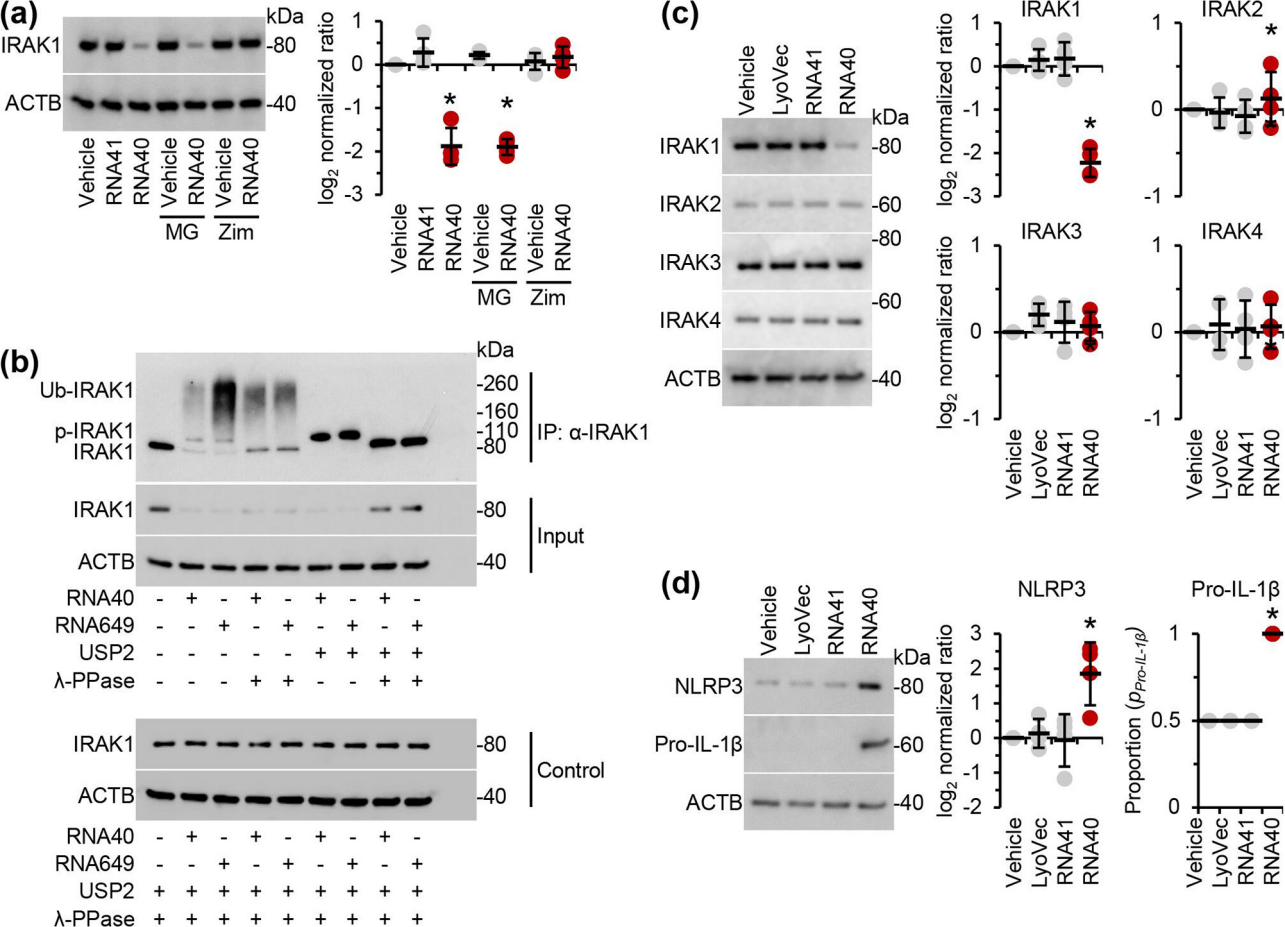
RNA40 induces phosphorylation and ubiquitination of IRAK1 in microglia. (**a**) MMG were pre-treated with 5 µM MG132 (MG) or 100 nM zimlovisertib (Zim) for 2 h, then treated for 24 h with 5 µg RNA40 ml^−1^ or controls. *Left*, representative western blots of IRAK1 and ACTB (the internal reference) from one biological replicate. *Right*, densitometric analysis of blots. Graphs represent the relative intensity of each target band to the corresponding ACTB signal. Each symbol represents an individual biological replicate (*n*=4); small horizontal lines indicate the means of four biological replicates±sd. (**b**) MMG were stimulated for 5 h with 5 µg RNA40 or RNA649 ml^−1^. IRAK1 was then immunoprecipitated (IP) from the cell extracts. A total of 30 µl immunoprecipitated IRAK1 was incubated with or without 50 U lambda protein phosphatase (λ-PPase) and/or 1.15 µg recombinant human ubiquitin specific peptidase 2 (USP2), before being analysed for IRAK1 content by western blot using an anti-IRAK1 antibody. Representative western blots of a single biological replicate are shown (*n*=4). Phosphorylated IRAK1 and phosphorylated and ubiquitinated IRAK1 are denoted by p-IRAK1 and Ub-IRAK1, respectively. (**c**) MMG were treated for 24 h with 5 µg RNA40 ml^−1^ or controls. *Left*, representative western blots of IRAK1, IRAK2, IRAK3, IRAK4, and ACTB (the internal reference) from one biological replicate. *Right*, densitometric analysis of blots. Graphs represent the relative intensity of each target band to the corresponding ACTB signal. Each symbol represents an individual biological replicate (*n*=4); small horizontal lines indicate the means of four biological replicates±sd. (**d**) MMG were treated for 24 h with 5 µg RNA40 ml^−1^ or controls. *Left*, representative western blots of pro-IL-1β, NLRP3, and ACTB (the internal reference) from one biological replicate. *Right*, densitometric analysis of blots. Graphs represent the relative intensity of each target band to the corresponding ACTB signal. Each symbol represents an individual biological replicate (*n*=4); small horizontal lines indicate the means of four biological replicates±sd. Statistical analyses were performed using two-tailed paired *t*-tests; *P<0.05.

Next, we examined whether IRAK1 was involved in the RNA40-induced TLR7 and TLR8-signalling pathways. Although IRAK1 activation requires neither phosphorylation nor ubiquitination [30, 36], recruitment and activation of IRAK1 at the myddosome can lead to its phosphorylation and ubiquitination, which results in the loss of IRAK1 detection by conventional western blotting [23, 47, 57–59]. Thus, we analysed this loss of detection via western blot in *TLR7* and/or *TLR8* silenced cells and controls. RNA40 treatment of MMG and control siRNA (siNS) transfected MMG induced the disappearance of IRAK1 from the blots indicating the phosphorylation and ubiquitination of IRAK1 (Fig. 1a). Although neither *TLR8* nor *TLR7* silencing resulted in the complete restoration of IRAK1, simultaneous silencing of *TLR7* and *TLR8* did, suggesting that, unlike in macrophages [16], RNA40 induces the activation of IRAK1 via both TLR7 and TLR8 in microglia (Fig. 1a). We confirmed that RNA40 did not induce the proteasomal degradation of IRAK1 using MG132, a proteasome and calpain inhibitor (Fig. 3a), and that it was indeed phosphorylated and ubiquitinated using an immunoprecipitation for IRAK1 after 5 h (as this is sufficient time for TLR signalling to have occurred) that we treated with a deubiquitinase (USP2) and/or a phosphatase (λ-PPase) that fully recovered the unmodified form of IRAK1 (Fig. 3b). Finally, we assessed whether the ubiquitination and phosphorylation of IRAK1 was dependent upon IRAK4 using zimlovisertib, a specific IRAK4 inhibitor. Notably, zimlovisertib restored the detection of IRAK1 on conventional western blots indicating that the phosphorylation and ubiquitination of IRAK1 via TLR8/TLR7 triggering was IRAK4-dependent (shown in Fig. 3a). Importantly, RNA40 had no effect on the expression of IRAK2, IRAK3, and IRAK4 (Fig. 3c), while also increasing the expression of both NLRP3 and pro-IL-β (Fig. 3d)

### Pacritinib inhibits RNA40-mediated cytokine production

**Fig. 4.**
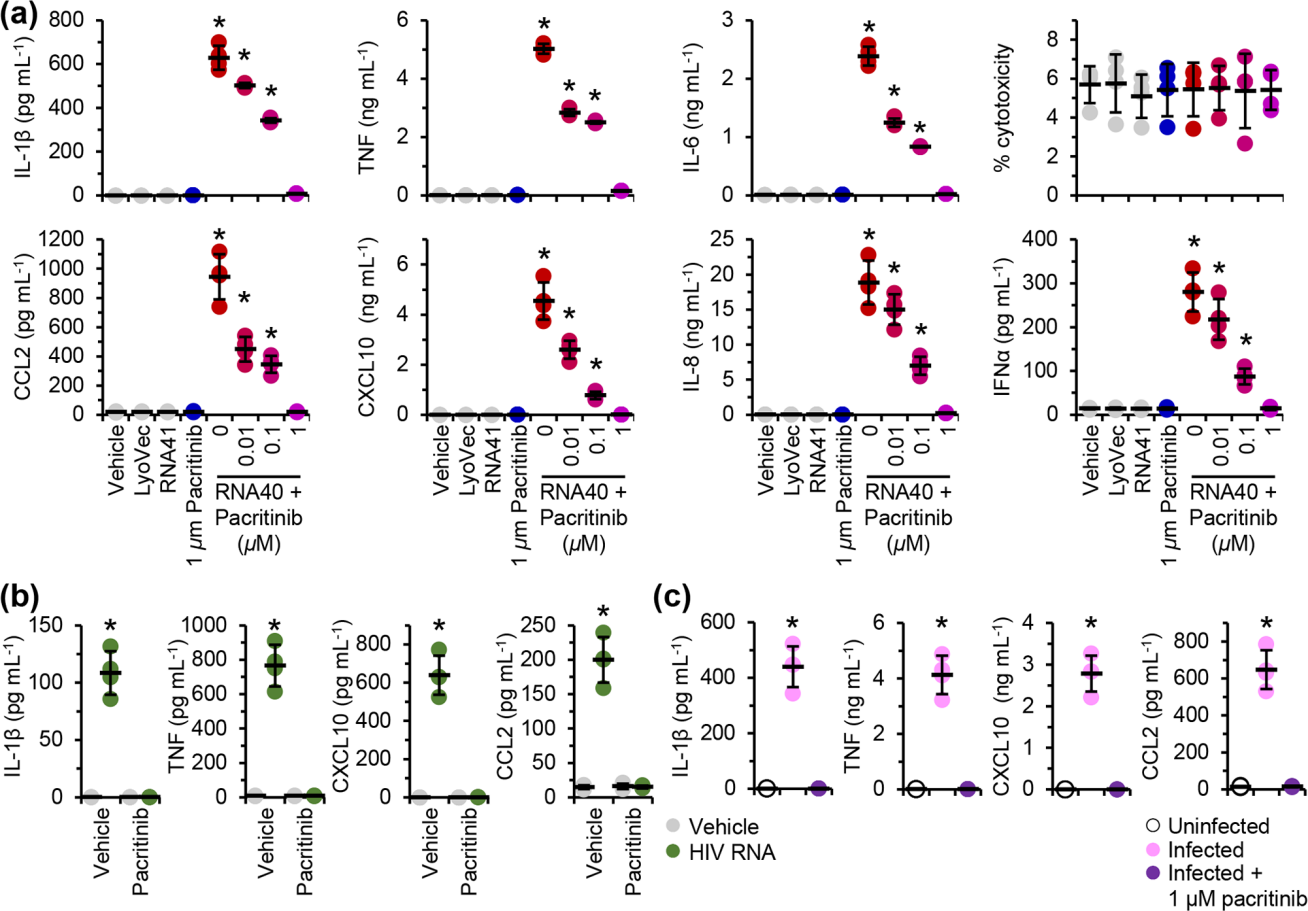
Pacritinib inhibits RNA40-induced pro-inflammatory cytokine and chemokine secretion from microglia. (**a**) MMG were pre-treated with pacritinib for 2 h before being treated for 24 h with 5 µg RNA40 ml^−1^. Cell supernatants were collected and analysed for cytokines and chemokines by ELISA, and LDH release using an LDH activity assay. (**b**) MMG were pre-treated with pacritinib for 2 h before being treated for 24 h with 1 µg HIV RNA ml^−1^. Cell supernatants were collected and analysed for cytokines and chemokines by ELISA. The experiment was performed with the same controls as in Fig. 2(a). (**c**) MMG from Fig. 2(b) were pre-treated with 5 µg plerixafor ml^−1^ and 5 µg maraviroc ml^−1^ for 24 h and 1 µM pacritinib for 2 h prior to co-culture with 293T cells expressing HIV Gag-iGFP_JRFL for a further 24 h in the presence of inhibitors. Cell supernatants were then analysed for cytokines and chemokines by ELISA. The experiment was performed with the same controls as in Fig. 2(d). Each symbol represents the mean of an individual biological replicate performed in triplicate; small horizontal lines indicate the grand means of four biological replicates±sd. Statistical analyses were performed using two-tailed paired *t-*tests; *P<0.05.

**Fig. 5.**
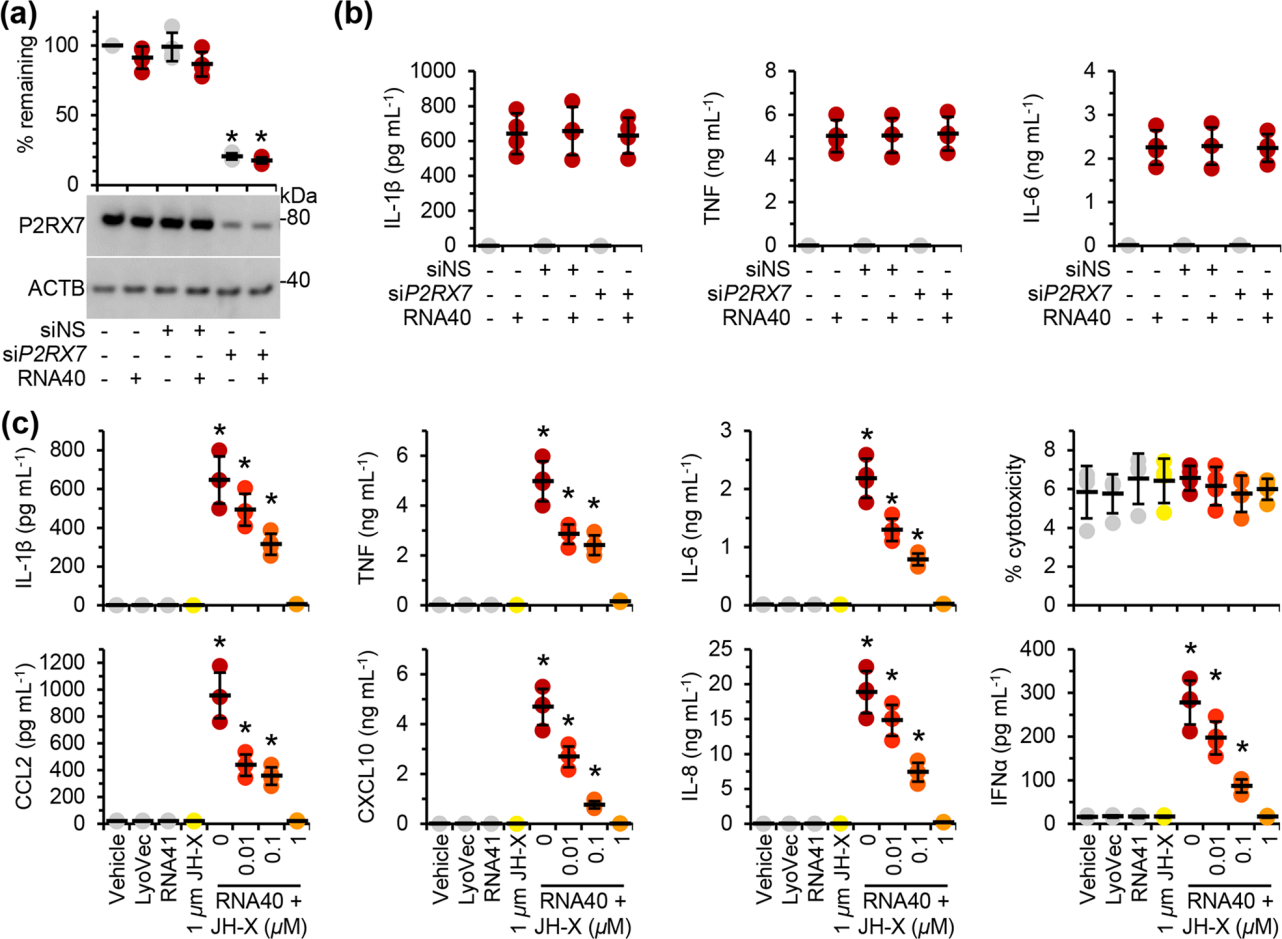
Pacritinib inhibits RNA40-mediated pro-inflammatory cytokine release from microglia release through the inhibition of IRAK1. (**a**) MMG were transfected with *P2RX7* (si*P2RX7*) or scrambled siRNA (siNS) for 48 h, then treated with 5 µg RNA40 ml^−1^ or control for 24 h. *Bottom*, representative western blots of P2RX7 and ACTB (the internal reference) from one biological replicate. *Top*, densitometric analysis of blots. Graphs represent the relative intensity of each target band to the corresponding ACTB signal. Each symbol represents an individual biological replicate (*n*=4); small horizontal lines indicate the means of four biological replicates±sd. (**b**) Cell supernatants from (**a**) were collected and analysed for cytokines by ELISA. Each symbol represents the mean of an individual biological replicate performed in triplicate; small horizontal lines indicate the grand means of four biological replicates±sd. (**c**) MMG pre-treated with JH-X-119-01 (JH-X) for 2 h were then treated for 24 h with 5 µg RNA40 ml^−1^. Cell supernatants were collected and analysed for cytokines and chemokines by ELISA, and LDH release using an LDH activity assay. Each symbol represents the mean of an individual biological replicate performed in triplicate; small horizontal lines indicate the grand means of four biological replicates±sd. Statistical analyses were performed using two-tailed paired *t*-tests; *P<0.05.

Having shown that IRAK1 is phosphorylated and ubiquitinated following TLR8 and TLR7 ligation in microglia, we next examined whether inhibition of IRAK1 using pacritinib would inhibit the RNA40 or HIV-induced expression and release of pro-inflammatory cytokines and chemokines. Pacritinib dose-dependently inhibited RNA40-induced (Fig. 4a), as well as the HIV RNA-induced (Fig. 4b) and endosomal HIV-induced (Fig. 4c) expression and release of pro-inflammatory cytokines and chemokines. As pacritinib also inhibits JAK2 [37], and JAK2 shares a consensus sequence with the purinergic receptor P2X 7 (P2RX7) [60] that plays a role in the ATP-induced IL-1β secretion from primary human monocytes [61], we silenced *P2RX7* (Fig. 5a). *P2RX7* silencing had no inhibitory effect on the RNA40-induced secretion of IL-1β, TNF, or IL-6 (Fig. 5b), indicating that pacritinib inhibition of RNA40-induced secretion of IL-1β is not due to inhibition of P2RX7, and that P2RX7 is not involved in the TLR7 or TLR8-mediated inflammasome activation in microglia. Finally, we also confirmed the role of IRAK1 inhibition using JH-X-119-01, a specific inhibitor of IRAK1 with a similar IC_50_ to pacritinib (9 nM) [62]. Like pacritinib, JH-X-119-01 dose-dependently inhibited the RNA40-induced expression and release of CCL2, CXCL10, IL-1β, IL-6, IL-8, IFNα, and TNF (Fig. 5c), suggesting that IRAK1 inhibition inhibited both TLR7 and TLR8 signalling cascades.

### Pacritinib inhibits the recruitment of the TAK1 complex to IRAK1

**Fig. 6.**
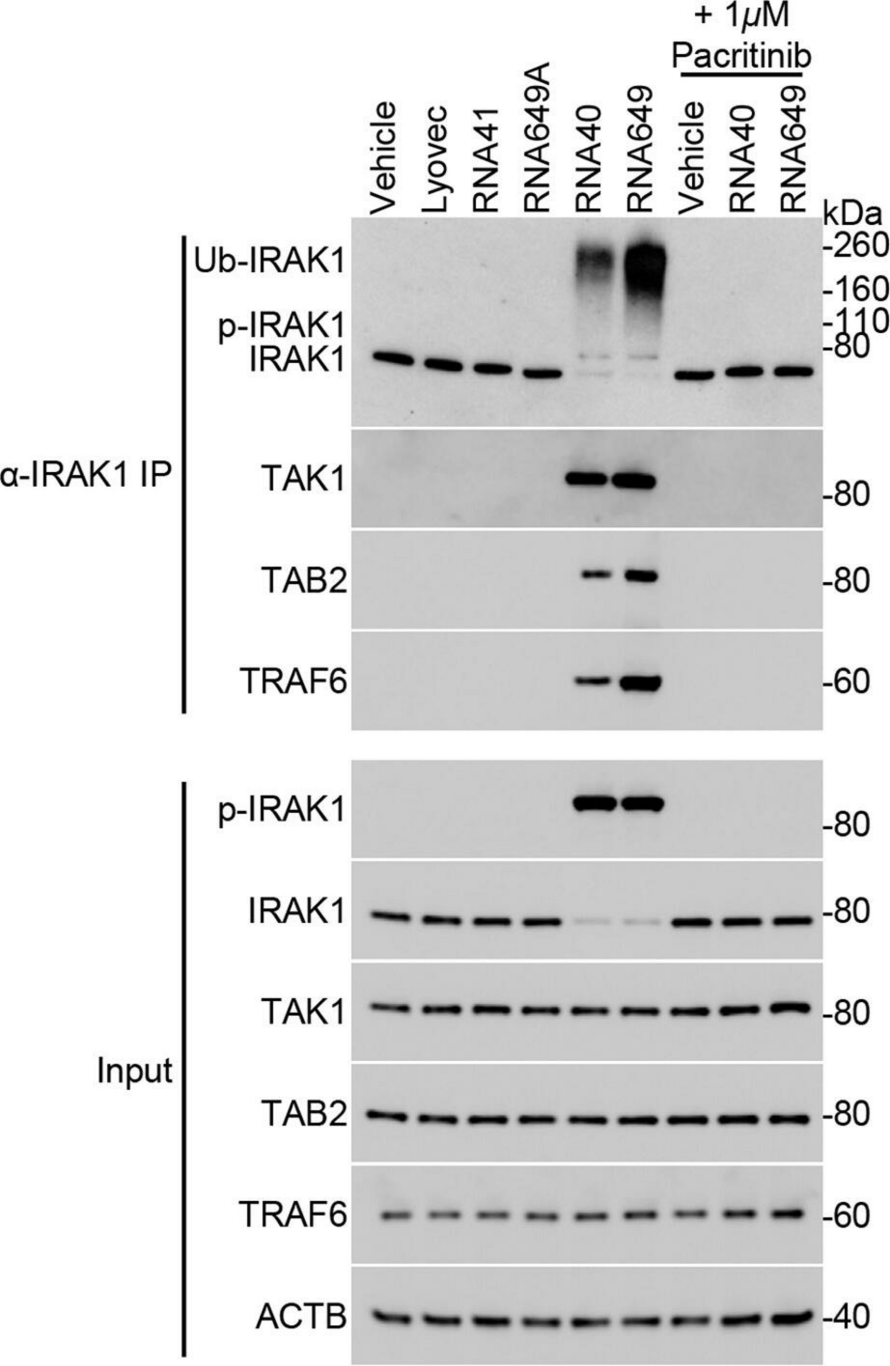
Pacritinib inhibits the association of IRAK1 with TRAF6 and the TAK1 complex in microglia. MMG were pre-treated with pacritinib or controls for 2 h, and then treated for 5 h with RNA40, RNA649 (another TLR8 agonist as a positive control), or controls (5 µg ml^−1^). Cells were lysed and IRAK1 was immunoprecipitated (IP). The presence of phosphorylated (p-IRAK1), ubiquitinated (Ub-IRAK1), and total IRAK1, TAK1, TAB2, TRAF6, and ACTB was assayed by western blotting. Representative western blots of a single biological replicate are shown (*n*=4).

As IRAK1 is ubiquitinated and phosphorylated in response to RNA40 exposure, we next investigated whether pacritinib inhibits these post-translational modifications using immunoprecipitation. Pacritinib completely inhibited the RNA40-induced phosphorylation and ubiquitination of IRAK1 (Fig. 6). As the phosphorylation and ubiquitination of IRAK1 are required for the recruitment of TRAF6 to IRAK1, and for the subsequent TAB2 and TAB3 recruitment to the IRAK1–TRAF6 complex that leads to the association of TAK1 with the IRAK1–TRAF6 complex, we examined the effect of IRAK1 inhibition on this complex formation. Pacritinib inhibited the association of TAK1, TAB2, and TRAF6 with IRAK1 in RNA40-treated MMG (Fig. 6).

**Fig. 7.**
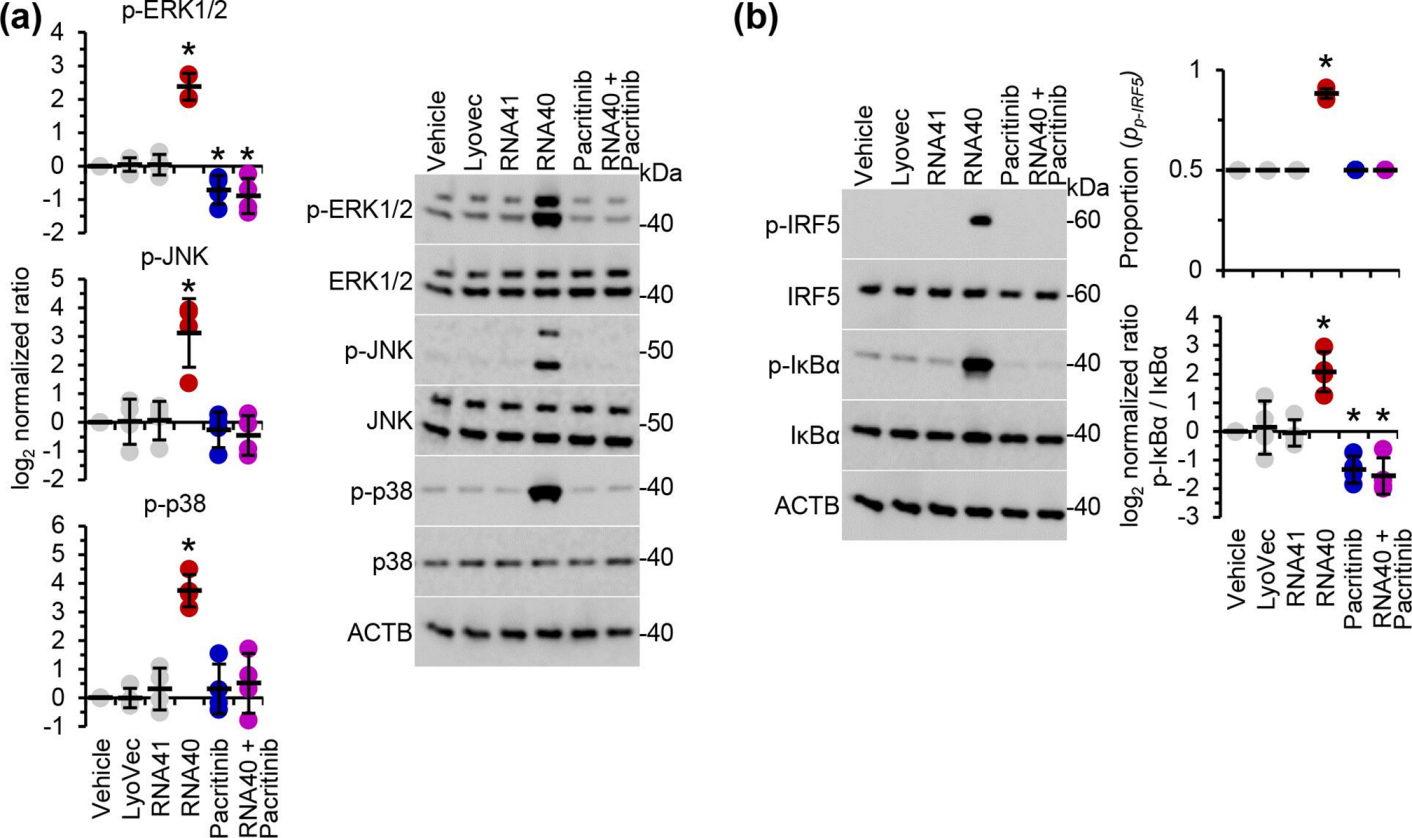
Pacritinib inhibits RNA40-mediated MAP kinase and NF-κB activation in microglia. MMG were pre-treated with pacritinib or controls for 2 h, and then treated for 6 h with 5 µg RNA40 ml^−1^. (**a**) *Right*, representative western blots of ERK1/2, JNK, and p38 and their phosphorylated forms (p-ERK1/2, p-JNK, and p-p38), and ACTB (the internal reference) from one biological replicate. *Left*, densitometric analysis of blots. Graphs represent the relative intensity of each target band to the corresponding ACTB signal. (**b**) *Left*, representative western blots of Ser^32^ phosphorylated IκBα, total IκBα, Ser^462^ phosphorylated IRF5, total IRF5, and ACTB (the internal reference) from one biological replicate. *Right*, densitometric analysis of blots. Graphs represent the relative intensity of each target band to the corresponding ACTB signal. Each symbol represents an individual biological replicate (*n*=4); small horizontal lines indicate the means of four biological replicates±sd. Statistical analyses were performed using two-tailed paired *t*-tests; *P<0.05.

The recruitment and activation of TAK1 leads to the phosphorylation and activation of mitogen-activated protein kinase kinase (MKK) 4 and MKK7 that phosphorylate and activate JNK1 [mitogen-activated protein kinase (MAPK) 8] and JNK2 (MAPK9). Moreover, the M1Ub ubiquitination of IRAK1 and subsequent interaction with NEMO leads to the phosphorylation and activation of IKKβ by TAK1. IKKβ then activates the mitogen-activated protein kinase kinase kinase 8 complex leading to the phosphorylation and activation of ERK1 (MAPK3), ERK2 (MAPK1), and p38α (MAPK14). Therefore, we evaluated whether pacritinib inhibited this MAP kinase activation cascade. At the concentration tested, pacritinib inhibition of IRAK1 arrested the downstream phosphorylation of ERK1, ERK2, JNK1, JNK2 and p38α in response to RNA40 (Fig. 7a).

IKKβ also activates two of the transcription factors normally required for inflammatory cytokine production, NF-κB and IRF5 (interferon regulatory factor 5). IKKβ activates and phosphorylates IRF5 at Ser^462^ [63], and activates NF-κB through the phosphorylation of the NF-κB inhibitory components (IκB), which releases NF-κB to translocate to the nucleus and initiate transcription [64]. Pacritinib completely inhibited the Ser^462^ phosphorylation of IRF5 and the Ser^32^ phosphorylation of IκBα in response to RNA40 treatment (Fig. 7b).

## Discussion

Despite ART-mediated HIV suppression, chronic inflammation and hyper-immune activation persists with increased levels of inflammatory biomarkers predictive of risk for comorbidities including NCI, as well as mortality [65, 66]. Chronic neuroinflammation can lead to the abnormal activation of microglia, a hallmark not only of HAND, but also of a diverse set of neurological diseases [67, 68] that can lead to neuronal apoptosis, the dysregulation of macroglia, and the loss of synaptodendritic signalling. Several studies have identified cerebrospinal fluid (CSF) inflammatory markers that correlate with the presence of HAND, among these, CCL2, CXCL10, IL-8, IFNα, and TNF are all significantly elevated in the CSF of PLWH with NCI and are thought to play a role in neuroinflammation and injury [69–73]. Thus, the suppression of neuroinflammation is of potential therapeutic benefit.

Despite the homogeneity between TLR7 and TLR8, the cellular distribution of these TLRs differs in humans, with TLR7 predominately expressed in plasmacytoid dendritic cells and B cells, and TLR8 primarily expressed in monocytes, macrophages and myeloid dendritic cells [74, 75]. However, both are expressed in human microglia [7]. Both TLR7 and TLR8 possess two ligand-binding sites – the first recognizes guanosine (in the case of TLR7) or uridine (for TLR8), while the second binds a uridine-containing oligoribonucleotide [76, 77]. Binding of ssRNA to the second site primes both receptors for the interaction with the nucleoside at the first site, leading to subsequent homodimer formation, the recruitment of the myddosome and downstream signalling [77, 78]. There are no consensus sequences for the uridine-containing oligoribonucleotide for either TLR, but some ssRNA sequence specificity has been shown for each of TLR7 and TLR8. ssRNAs longer than a 3-mer containing uridine at a non-terminal position can bind and activate TLR7, while any uridine-containing oligonucleotide longer than a 2-mer and containing a purine base is sufficient to bind and activate TLR8 [76–78]. In the present study, we found that RNA40 is sufficient to activate both TLR7 and TLR8 signalling cascades in human microglia that leads to a pro-inflammatory response, which includes the expression of CCL2, CXCL10, IFNα, IL-1β, IL-6, IL-8, and TNF. However, these data show that there are differences in the TLR required to trigger the production of these cytokines and chemokines. CCL2, IFNα, IL-6, and TNF are equally induced by either TLR7 or TLR8, IL-1β and IL-8 are dependent upon TLR8 only, and the expression of CXCL10 is dependent upon TLR7 – these differences warrant future investigation. It is possible that the increased expression of IL-1β and IL-8 in *TLR7* silenced cells is because TLR7 and TLR8 share many adaptor proteins, and that silencing the expression of one receptor enhances accessory protein availability to the other. As dual *TLR7* and *TLR8* silencing was necessary to completely inhibit the pro-inflammatory response, antagonists of either TLR7 or TLR8 may be insufficient to obtain a beneficial effect. IRAK1 inhibition overcomes this and completely blocks the RNA40-mediated pro-inflammatory response as demonstrated here using pacritinib and JH-X-119-01.

Unchecked TLR signalling cascades are involved in the progression of many inflammatory disorders, including autoimmune diseases, cardiovascular diseases, fibrotic diseases, and sepsis, as well as in the initiation and progression of solid tumour and haematological malignancies. Moreover, although TLR activity is essential for critical brain functions such as synaptogenesis and neurogenesis [79–81], increasing evidence also supports aberrant TLR signalling in the pathogenesis of major neurodegenerative diseases including Alzheimer’s disease, Parkinson’s disease, multiple sclerosis and vascular dementia, as well as in traumatic brain injuries, and alcohol and chemotherapy-induced NCI [82]. As current treatments for HIV NCIs only attenuate the symptoms, elucidating any potential mechanism(s) of TLR-mediated neuroinflammation in the CNS of PLWH may be important for the development of therapeutics to prevent NCI not only in HAND, but also in other neurodegenerative diseases. Indeed, the targeting of TLRs and their downstream signalling pathways as a potential therapeutic approach has already demonstrated efficacy in animal models of several neurodegenerative diseases, including Alzheimer’s disease and Parkinson’s disease, by suppressing microglia activation and neuroinflammation [83–85]. As IRAK1 is a key component of both IL-1R and TLR-mediated signalling, the pharmacological inhibition of IRAK1 thus has potential therapeutic applications in neurocognitive diseases. Additionally, as pacritinib also inhibits JAK2, it may also suppress HIV transcription [86]. However, the neuropenetrative ability of pacritinib, and indeed any drug targeting the CNS, is of undoubted importance. In this regard, the nanoencapsulation and targeting of pacritinib to the CNS is of interest. While the inhibition of IRAK1 does raise concerns regarding immunosuppression and secondary infections, clinical trials of pacritinib for autoimmune diseases, fibrotic diseases, and oncological diseases have shown acceptable safety profiles [87], and pacritinib has been FDA approved in the USA for the treatment of myelofibrosis marked by severe thrombocytopaenia.

In summary, our data demonstrate that IRAK1 inhibition inhibits the TLR7 and TLR8-mediated pro-inflammatory response to HIV RNA. These data suggest that drugs targeting TLRs, and their downstream signalling cascades, should be considered as potential therapeutic approaches for HIV-associated neurological disorders and neurodegenerative diseases.
